# Targeted cytotoxic analogue of bombesin/ gastrin-releasing peptide inhibits the growth of H-69 human small-cell lung carcinoma in nude mice

**DOI:** 10.1038/sj.bjc.6690794

**Published:** 1999-11

**Authors:** H Kiaris, A V Schally, A Nagy, B Sun, P Armatis, K Szepeshazi

**Affiliations:** Endocrine, Polypeptide and Cancer Institute, Veterans Affairs Medical Center, 1601 Perdido Street; Section of Experimental Medicine, Department of Medicine, Tulane University School of Medicine, New Orleans, LA 70112, USA

**Keywords:** tumour inhibition, cancer therapy, hormone analogues, tumour targeting, doxorubicin, bombesin receptor, hGRPR, hNMBR, RT-PCR

## Abstract

Recently, we developed a powerful cytotoxic analogue of bombesin AN-215, in which the bombesin-like carrier peptide Gln–Trp–Ala–Val–Gly–His–Leu–Ψ(CH_2_-NH)–Leu–NH_2_ (RC-3094) is conjugated to a potent derivative of doxorubicin, 2-pyrrolinodoxorubicin (AN-201). Small-cell lung carcinomas (SCLCs) are known to express high levels of bombesin receptors. We evaluated whether these receptors could be used for targeting cytotoxic bombesin analogue to H-69 SCLC cells. H-69 cells were xenografted into male nude mice, which then received an intravenous injection of AN-215, cytotoxic radical AN-201, the carrier peptide RC-3094 alone or unconjugated mixture of RC-3094 and AN-201. The levels of mRNA for bombesin receptor subtypes were evaluated by reverse transcription-polymerase chain reaction. In vitro, both the analogue AN-215 and the radical AN-201 showed strong antiproliferative effects on H-69 cells, AN-215 requiring more time to exert its action at 10^–8^M concentration than AN-201. In vivo, the growth of H-69 SCLC tumours was significantly inhibited by the treatment with 200 nmol kg^–1^ of AN-215, while equimolar doses of the cytotoxic radical AN-201 or the mixture of AN-201 and the carrier peptide were toxic and produced only a minor tumour inhibition as compared with control groups. mRNA for bombesin receptor subtypes 2 (BRS-2) and 3 (BRS-3) was detected in H-69 tumours. The mRNA levels for BRS-3, but not for BRS-2, were lower in the AN-215-treated tumours as compared with controls. Our results demonstrate that the cytotoxic bombesin analogue AN-215 could be considered for targeted therapy of tumours, such as SCLC, that express bombesin receptors. © 1999 Cancer Research Campaign


*British Journal of Cancer* (1999) **81**(6), 966–971
© 1999 Cancer Research Campaign
Article no. bjoc.1999.0794

### Targeted cytotoxic analogue of bombesin/gastrin-releasing peptide inhibits the growth of H-69 human small-cell lung carcinoma in nude mice

**H Kiaris, AV Schally, A Nagy, B Sun, P Armatis and K Szepeshazi**

Endocrine, Polypeptide and Cancer Institute, Veterans Affairs Medical Center, 1601 Perdido Street and Section of Experimental Medicine, Department of Medicine, Tulane University School of Medicine, New Orleans, LA 70112, USA

**Summary** Recently, we developed a powerful cytotoxic analogue of bombesin AN-215, in which the bombesin-like carrier peptide Gln–Trp–Ala–Val–Gly–His–Leu–Ψ($CH_2$-NH)–Leu–$NH_2$ (RC-3094) is conjugated to a potent derivative of doxorubicin, 2-pyrrolinodoxorubicin (AN-201). Small-cell lung carcinomas (SCLCs) are known to express high levels of bombesin receptors. We evaluated whether these receptors could be used for targeting cytotoxic bombesin analogue to H-69 SCLC cells. H-69 cells were xenografted into male nude mice, which then received an intravenous injection of AN-215, cytotoxic radical AN-201, the carrier peptide RC-3094 alone or unconjugated mixture of RC-3094 and AN-201. The levels of mRNA for bombesin receptor subtypes were evaluated by reverse transcription-polymerase chain reaction. In vitro, both the analogue AN-215 and the radical AN-201 showed strong antiproliferative effects on H-69 cells, AN-215 requiring more time to exert its action at $10^{-8}$ M concentration than AN-201. In vivo, the growth of H-69 SCLC tumours was significantly inhibited by the treatment with 200 nmol $kg^{-1}$ of AN-215, while equimolar doses of the cytotoxic radical AN-201 or the mixture of AN-201 and the carrier peptide were toxic and produced only a minor tumour inhibition as compared with control groups. mRNA for bombesin receptor subtypes 2 (BRS-2) and 3 (BRS-3) was detected in H-69 tumours. The mRNA levels for BRS-3, but not for BRS-2, were lower in the AN-215-treated tumours as compared with controls. Our results demonstrate that the cytotoxic bombesin analogue AN-215 could be considered for targeted therapy of tumours, such as SCLC, that express bombesin receptors. © 1999 Cancer Research Campaign

**Keywords:** tumour inhibition; cancer therapy; hormone analogues; tumour targeting; doxorubicin; bombesin receptor; hGRPR; hNMBR; RT-PCR

Lung cancer is the leading cause of cancer-related deaths in the Western world. Small-cell lung carcinoma (SCLC) accounts for about 20% of all cases of lung cancer (Ihde, 1995). Although the introduction of modern chemotherapeutic agents and improved techniques in radiotherapy increased the survival rate of SCLC patients, there is a continued need for the development of novel therapeutic strategies. The use of targeted cytotoxic analogues represents a modern approach to the treatment of various cancers because it may improve tumour inhibition and decrease the toxicity (Schally and Nagy, 1999). Typically, targeted agents consist of a toxic moiety conjugated to a ligand possessing a high affinity for tumour cells. Certain molecules such as enzymes, antigens, or receptors that are expressed predominantly on the surface of cancer cells may be used as targets to increase the selectivity of therapy (Pastan and FitzGerland, 1991).

SCLC is a neuroendocrine tumour. Many SCLC tumours and cell lines express high levels of bombesin-like peptides and/or specific high-affinity binding sites for bombesin. An autocrine role for bombesin-like peptides in stimulating the growth of some SCLC tumours has been suggested (Cuttitta et al, 1985; Moody et al, 1985; Moody and Cuttitta, 1993; Pinski et al, 1994; Koppán et al, 1998). Chronic treatment with bombesin antagonists significantly inhibits the proliferation of SCLC in vivo and in vitro (Pinski et al, 1994; Koppán et al, 1998). Molecular cloning revealed the presence of three distinct bombesin receptors in humans. Bombesin receptor subtype-1 (hGRPR/BRS-1) binds gastrin-releasing peptide (GRP), and hNMBR/BRS-2 is preferentially activated by neuromedin B (Von Schrenck et al, 1989; Spindel et al, 1993). BRS-3 is considered an orphan receptor because its natural ligand remains unknown (Fathi et al, 1993). The existence of a fourth subtype of bombesin receptor (BRS-4) has also been predicted in mammals, after the identification of BRS-4 in frogs (Nagalla et al, 1995). Bombesin receptors belong to the superfamily of the seven-transmembrane domain receptors and are involved in signal transduction pathways by hydrolysing GTP (Spindel et al, 1993) and stimulating the expression of c-*fos* and c-*jun* mRNAs (Draoui et al, 1994).

Considering that bombesin receptors have been detected in approximately 95% of SCLCs (Toi-Scott et al, 1996), these binding sites could be used for targeting of cytotoxic bombesin analogues. Thus, we recently developed a cytotoxic analogue of bombesin AN-215 (Nagy et al, 1997), which consists of a potent cytotoxic derivative of doxorubicin (DOX), 2-pyrrolino-DOX (AN-201) (Nagy et al, 1996), covalently linked to the carrier octapeptide RC-3094 (Gln–Trp–Ala–Val–Gly–His–Leu–Ψ($CH_2$-NH)–Leu–$NH_2$) (Nagy et al, 1997). AN-215 shows high affinity to bombesin receptors on Swiss 3T3 cells and it retains the antiproliferative effect of its cytotoxic moiety (Nagy et al, 1997).

NCI-H-69 cell line is an important model of SCLC and when xenografted into nude mice expresses bombesin receptors. In this

*Received 29 January 1999*
*Revised 4 May 1999*
*Accepted 5 May 1999*

*Correspondence to:* AV Schally study we evaluated the antiproliferative activity of the cytotoxic bombesin analogue AN-215 in vivo and in vitro.

### MATERIALS AND METHODS

### Peptides and cytotoxic radical

Cytotoxic radical 2-pyrrolino-DOX (AN-201), the bombesin-like carrier analogue Gln–Trp–Ala–Val–Gly–His–Leu–Ψ($CH_2$-NH)–Leu–$NH_2$ (RC-3094) and the cytotoxic bombesin analogue 2-pyrrolino-DOX-14-*O*-hemiglutarate linked to the amino terminal of Gln–Trp–Ala–Val–Gly–His–Leu–Ψ($CH_2$-NH)–Leu–$NH_2$ (AN-215) were synthesized in our laboratory as described (Nagy et al, 1997). Before the intravenous (i.v.) injection, the compounds were dissolved in 5% (w/v) aqueous D-mannitol solution (Sigma, St Louis, MO, USA).

### Cell lines

The human SCLC cell line NCI-H-69 was obtained from American Type Culture Collection (ATCC, Manassas, VA, USA) and cultured in RPMI-1640 medium supplemented with 10% fetal bovine serum (FBS), 2 mM glutamine, penicillin (100 U $ml^{-1}$), streptomycin (100 μg $ml^{-1}$) and amphotericin B (100 U $ml^{-1}$) at 37°C in a humidified 95% air/5% carbon dioxide atmosphere. Cells were passaged weekly and routinely monitored for mycoplasma contamination using a detection kit (Boehringer Mannheim, Mannheim, Germany). All culture media components were purchased from Gibco (Grand Island, NY, USA).

### MTT assay

The assay was performed as described (Plumb, 1989). Briefly, cells were seeded into 96-well microplates and cultured for 18 h. AN-215 and AN-201 were added to the medium in final concentrations of $10^{-9}$–$10^{-7}$ M. Control cultures received HITES medium alone. HITES medium contained RPMI-1640 plus 4 mM glutamine, 10 mM HEPES (Gibco, Grand Island, NY, USA), 5 μg $ml^{-1}$ insulin, 10 μg $ml^{-1}$ transferrin, 30 nM sodium selenide, 10 nM hydrocortisone, 10 nM β-oestradiol, all from Sigma (St Louis, MO, USA). After 72 h of culture, the medium was removed and 200 μl HITES containing 80 μg MTT [3(4,5-dimethylthiazol-2yl)-2,5-diphenyl tetrazolium bromide; Sigma] was added. The microplates were incubated for 4 h at 37°C in darkness. The medium was removed, cells were washed twice with RPMI-1640 and 200 μl dimethyl sulphoxide (DMSO; Sigma) was added followed by 25 μl of Sorensen's glycine buffer (0.1 M glycine plus 0.1 M sodium chloride, pH 10.5). After a brief mixing, the plates were read at 540 nm on the plate reader (Beckman, Palo Alto, CA, USA). Results were calculated as % T/C, where T = optical density ($OD_{540\ nm}$) of treated cultures (HITES medium plus AN-201 or AN-215) and C = $OD_{540\ nm}$ of untreated cultures (HITES medium alone).

### Animals

Five- to 6-week-old male athymic (Ncr *nu/nu*) nude mice were obtained from National Cancer Institute (NCI; Bethesda, MD, USA). The mice were housed in sterile cages under laminar flow hoods in a temperature-controlled room with a 12-h light/12-h dark schedule and were fed autoclaved chow and water ad libitum. Their care was in accord with institutional guidelines.

### Experimental protocol

NCI-H-69 cells growing exponentially were implanted into three male nude mice by subcutaneous (s.c.) injection of $10^7$ cells in the right flank. Tumours resulting after 8 weeks in donor animals were aseptically dissected and mechanically minced. Three $mm^3$ pieces of tumour tissue were transplanted (s.c.) by trocar needle into 50 mice under methoxyflurane (Metofane, Pittman-Moore, Mundelein, IL, USA) anaesthesia. Three weeks after transplantation, when tumours reached a volume of approximately 45 $mm^3$, mice were randomized and divided into five experimental groups of 5–7 animals each, which received the following treatments as a single i.v. injection: group 1, control, vehicle solution (5% mannitol); group 2, cytotoxic radical AN-201 at a dose of 200 nmol $kg^{-1}$; group 3, unconjugated mixture of the cytotoxic radical AN-201 and the carrier RC-3094 at a dose of 200 nmol $kg^{-1}$; group 4, cytotoxic analogue AN-215 at a dose of 200 nmol $kg^{-1}$; group 5, carrier peptide RC-3094 at a dose of 200 nmol $kg^{-1}$. All experiments were approved by the institutional ACUC and the procedures were essentially in accordance with UKCCCR guidelines (1998) for the welfare of animals in experimental neoplasia.

### Histological methods

Tumour samples were processed as described (Szepeshazi et al, 1992). The extent of necrosis was evaluated with the point-counting method on tumour slides stained with haematoxylin and eosin (H&E). For the measurement of the number of mitotic and apoptotic cells, ten high-power fields were considered and the numbers of mitotic and apoptotic cells per 1000 cells (mitotic and apoptotic indices) were calculated in H&E-stained slides.

### RNA extraction

Total RNA was isolated using the RNAzol B reagent (TEL-TEST, Inc., Friendswood, TX, USA) following the manufacturer's instructions. The quantity and the quality of the RNA was assessed by spectrophotometry at 260 nm and 280 nm ($O.D._{260}/O.D._{280} > 1.8$).

### Reverse transcription

One microgram of total RNA was added in a test tube containing 10 mM Tris–HCl (pH 8.3), 50 mM potassium chloride (KCl), 5 mM magnesium chloride ($MgCl_2$), 1 mM each of deoxyribonucleoside triphosphate, 2.5 μM random hexamers, 1 U RNAase inhibitor and double-distilled water in a final volume of 19 μl. Following heating for 10 min at 65°C and quenching on ice, 2.5 U Moloney murine leukaemia virus reverse transcriptase (Perkin-Elmer, Norwalk, CT, USA) were added and the reaction mixture was incubated for 10 min at room temperature following incubation at 42°C for 1 h. The reaction was terminated by heating at 95°C for 5 min and quenching on ice.

### Polymerase chain reaction (PCR) amplification

One microlitre of the cDNA was amplified in a 50 μl solution containing 10 mM Tris–HCl (pH 8.3), 50 mM KCl, 1.5 mM $MgCl_2$, 200 μM of each dNTP, 2.5 U *Taq* DNA polymerase (Perkin-Elmer, Norwalk, CT, USA) and 0.4 mM of each primer. The primers used were 5′-TCCTCTGACTTCAACAGCGACACC-3′ and

© 1999 Cancer Research Campaign 

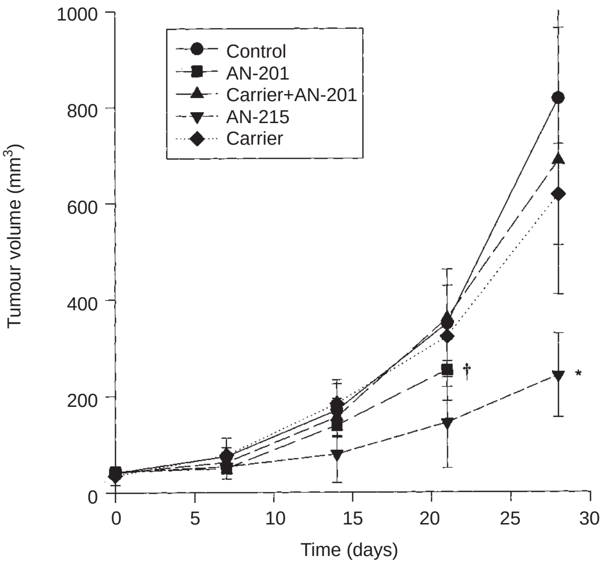

**Figure 1** Changes in tumour volume in athymic nude mice bearing subcutaneous xenografts of H-69 SCLC after treatment with 200 nmol kg$^{-1}$ of cytotoxic bombesin analogue AN-215, cytotoxic radical AN-201, unconjugated mixture of AN-201 and the carrier peptide RC-3094, or the carrier peptide RC-3094. Vertical bars show s.e.m. **P* < 0.05 vs control. †Three mice in the AN-201-treated group died during the fourth week of treatment and the volume of tumours recorded on day 21 is shown

5′-TCTCTCTTCCTCTTGTGCTCTTGG-3′ for human glyceraldehyde-3-phosphate dehydrogenase (hGAPDH), 5-ATTTGGCAGGATTGGCTGC-3′ and 5′-TGAGGCAGATCTTCATCAG-3 for BRS-1 (GRP receptor, GRPR), 5′-CGGACTCTGCTGGAAAGGA-3′ and 5′-GACGTCTGCATGTCCATGG-3 for BRS-2 (neuromedin B receptor, hNMBR) and 5′-GCTCTGTGGTTTCTAACG-3′ and 5′-CTGCCTTGTATCTGTCAGC-3′ for BRS-3 (Sun and Schally, in preparation). Samples were denatured at 94°C for 5 min and then subjected to 40 cycles of 94°C for 1 min (denaturation), 52°C for 1 min (annealing) and 72°C for 1 min (extension) for BRS-1 and BRS-3, 94°C for 30 s, 62°C for 30 s and 72°C for 30 s for BRS-2 or 26 cycles of 94°C for 35 s, 58°C for 40 s and 72°C for 40 s for hGAPDH, followed by a final extension at 72°C for 5 min using a Perkin-Elmer DNA thermal cycler model 2400. The number of cycles was determined in preliminary experiments to be within the exponential range of PCR amplification. Five microlitres of each PCR product were electrophoresed in an 8% polyacrylamide gel, stained with silver and quantified densitometrically using a scanning densitometer (Model GS-700, BioRad, CA, USA) coupled with the BioRad PC analysis software. The mRNA levels of each bombesin receptor subtype were normalized versus the corresponding levels of hGAPDH.

### Statistical analysis

The data are expressed as the mean ± s.e.m. Statistical evaluation of data was performed by Student's *t*-test (two-tailed) and Duncan's multiple range test. The SigmaPlot computer software (Jandel, San Rafael, CA, USA) was used for preparation of figures.

### RESULTS

### Anti-tumour effects and toxicity

Administration of a single i.v. injection of cytotoxic bombesin analogue AN-215 produced a significant ($P < 0.05$) growth inhibition of NCI-H-69 SCLC xenografted into nude mice (Figure 1). Twenty-eight days after the injection the tumour volume in the animals treated with AN-215 was significantly reduced to 242.2 ± 86.9 mm$^3$ ($P < 0.05$) (Table 1) as compared with the control group which measured 817.9 ± 206.5 mm$^3$. Tumour inhibition produced by the treatment with an equimolar dose of AN-201 (Figure 1 and Table 1) was not significant and accompanied by high toxicity; five of six animals were dead in the AN-201-treated group, while only one of six mice died in the AN-215-treated group before the termination of the experiment. A high toxicity was also found in the group that received the unconjugated mixture of AN-201 and carrier RC-3094, five of seven mice being dead 28 days after the treatment. Administration of the carrier peptide was not toxic, but had no significant tumour inhibitory effects. A significant loss of body weight occurred in all groups injected with cytotoxic agents. The reduction in body weight produced by AN-215 was smaller and of a shorter duration than that caused by radical AN-201. The body weights in the surviving animals recovered within 2 weeks after the injection (Figure 2).

### Histology

The quantitative histological data are shown in Table 2. There was an increase in the apoptotic index and the percentage area of

**Table 1** Effect of a single intravenous injection of 200 nmol kg$^{-1}$ cytotoxic bombesin analogue AN-215, cytotoxic radical AN-201, carrier peptide RC-3094 and mixture of RC-3094 and AN-201 on tumour volume, tumour weight and animal mortality in athymic nude mice bearing H-69 SCLC, recorded 28 days later

| Substance | Initial tumour volume (mm$^3$) | Final tumour volume (mm$^3$) | Tumour burden (mg g$^{-1}$ body weight) | Number of deaths/ number of all animals |
|---|---|---|---|---|
| Control | 42.3 ± 7.8 | 817.9 ± 206.5 | 31.2 ± 7.2 | 0/6 |
| AN-201 | 43.4 ± 6.11 | [a] | [a] | 5/6[b] |
| AN-201 + RC-3094 | 40.7 ± 7.3 | 687.1 ± 276.8 | 30.2 ± 7.9 | 5/7 |
| AN-215 | 38.2 ± 4.8 | 242.2 ± 86.9* | 10.5 ± 3.7 | 1/6 |
| RC-3094 | 35.4 ± 8.9 | 616.8 ± 103.3 | 22.7 ± 1.7 | 0/5 |

[a]Four mice were alive on day 21, but 3 died before day 26 and the volume of tumours recorded on day 21 was 253 ± 63.2. The only surviving mouse was sacrificed on day 26 and thus no data on the tumour burden are available. [b]Deaths until day 26 when the last surviving animal was sacrificed (see also footnote a). **P* < 0.05 vs controls.

 © 1999 Cancer Research Campaign

**Table 2** Effect of treatment with cytotoxic bombesin analogue AN-215 on some histological characteristics of H-69 SCLC xenografted into nude mice

| Substance | % area of necrosis in tumours | Mitotic index | Apoptotic index | Ratio of apoptotic to mitotic indices |
|---|---|---|---|---|
| Control | 18.1 ± 7.0 | 8.65 ± 0.35 | 7.0 ± 2.0 | 0.82 ± 0.26 |
| AN-215 | 27.4 ± 10.9 | 11.4 ± 0.10 | 11.5 ± 2.1 | 1.01 ± 0.18 |

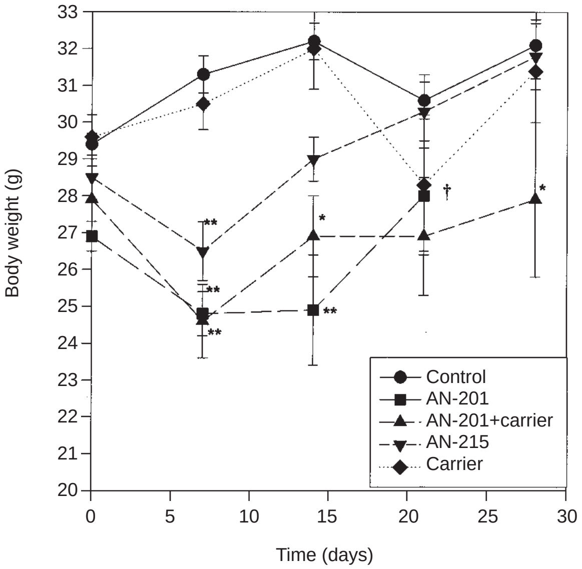

**Figure 2** Changes in body weight of athymic nude mice bearing subcutaneous xenografts of H-69 SCLC after treatment with 200 nmol kg$^{-1}$ of cytotoxic bombesin analogue AN-215, cytotoxic radical AN-201, unconjugated mixture of AN-201 and the carrier peptide RC-3094, or the carrier peptide RC-3094. Vertical bars show s.e.m. **P* < 0.05 vs control, ***P* < 0.005. †Three mice in the AN-201 treated-group died during the fourth week of treatment and the body weight recorded on day 21 is shown necrosis in tumours of the mice treated with AN-215 as compared with the tumours in the control group. Although the mitotic index was elevated in the tumours of the AN-215-treated mice, the ratio of apoptotic to mitotic indices remained higher than in the controls. However, the differences between the tumours from the control and the AN-215-treated mice were not statistically significant, probably because the cytotoxic agent was administered 28 days before the histological analysis was performed.

### Antiproliferative effect in vitro

The antiproliferative effects produced by the cytotoxic radical AN-201 and the cytotoxic bombesin analogue AN-215 in H-69 cells cultured in vitro were assessed by the MTT assay. H-69 cells were exposed to the cytotoxic agents at various concentrations for periods of 1 min to 24 h. AN-201 and AN-215 had no effect at $10^{-9}$ M, while at $10^{-7}$ M these cytotoxic agents had similar antiproliferative activity (Figure 3A). However, when H-69 cells were exposed to the compounds at a concentration of $10^{-8}$ M, AN-215 required more time than AN-201 to produce a comparable inhibitory effect (Figure 3B). After 24 h, however, their antiproliferative effect was no longer discernible (Figure 3B).

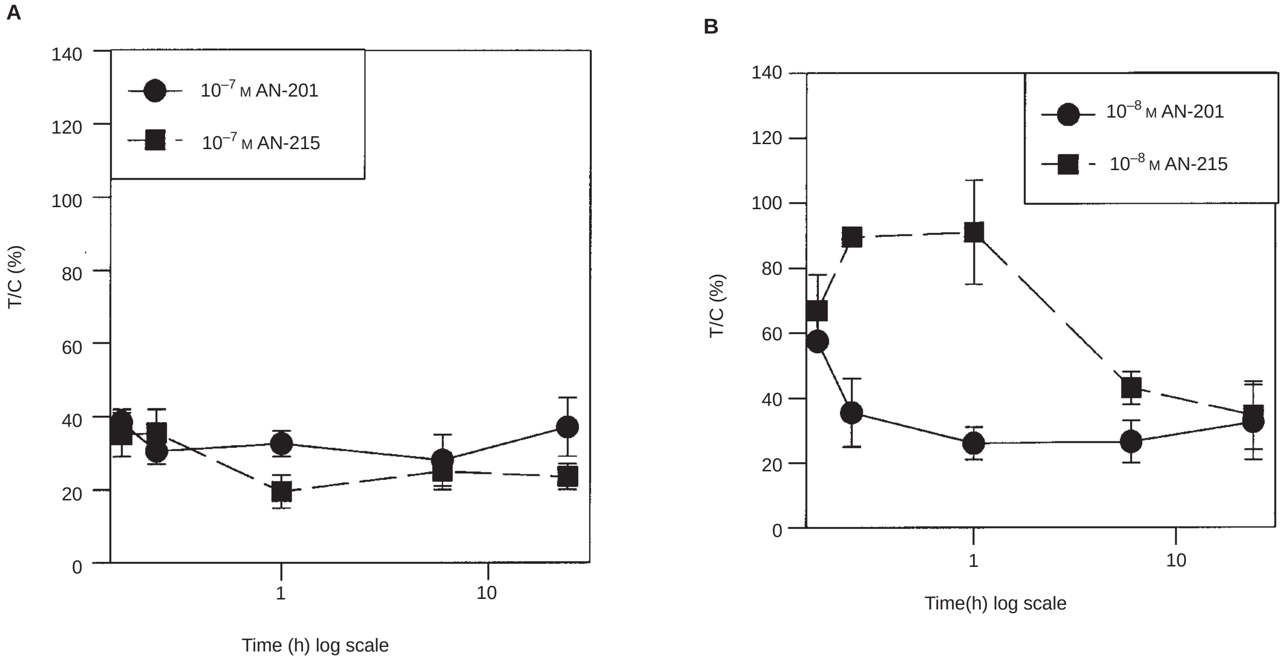

**Figure 3** Effect of cytotoxic bombesin analogue AN-215 and cytotoxic radical AN-201 at $10^{-7}$ M (**A**) and $10^{-8}$ M (**B**) on the growth of H-69 SCLC in vitro. Results are expressed as the percentage of the number of cells found after the treatment with the cytotoxic agents compared to the number of the untreated cells. Vertical bars show s.e.m. from two independent experiments

*© 1999 Cancer Research Campaign* 

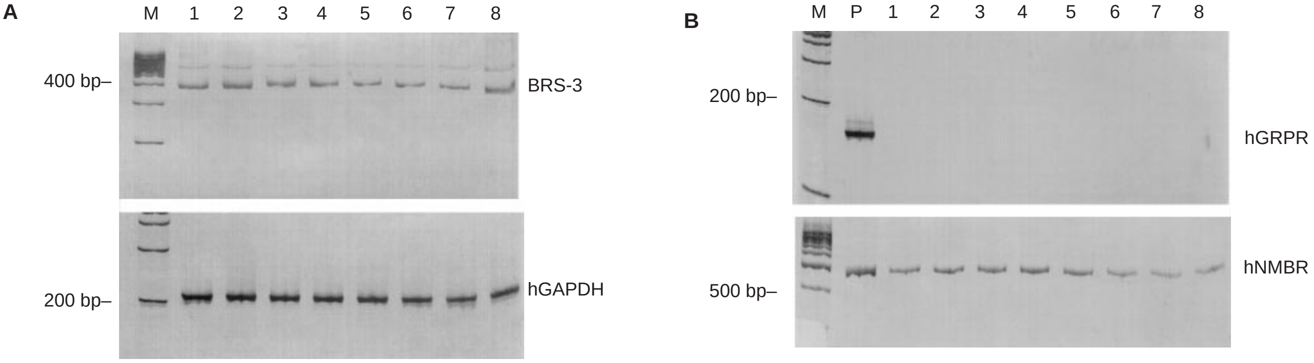

**Figure 4** Polyacrylamide gel electrophoresis of reverse-transcribed and subsequently PCR-amplified mRNAs for BRS-3 (**A**) and hGRPR/BRS-1 and hNMBR/BRS-2 (**B**) in H-69 SCLC xenografted into nude mice. The bands for corresponding levels of hGAPDH are also shown (**A**). M, molecular weight DNA marker; P, positive control; 1–4, untreated tumours; 5–8, AN-215-treated tumours. All PCR amplification reactions yielded products of the expected size which were 158 bp for BRS-1, 484 bp for BRS-2, 375 bp for BRS-3 and 207 bp for hGAPDH. No expression of BRS-1 was detected, but the mRNA for BRS-2 and BRS-3 were identified.

### Expression of mRNA for bombesin receptors

The levels of mRNA for bombesin receptor subtypes hGRPR/BRS-1, hNMBR/BRS-2 and BRS-3 were analysed in H-69 SCLC tumours of control and AN-215-treated animals (Figure 4). Four tumours from each group were used for the analyses. mRNA for hGRPR/BRS-1 was not found in the control or the AN-215-treated tumours, but the presence of mRNA for BRS-2 and BRS-3 was detected. Densitometric analyses of RT-PCR products revealed that hNMBR/BRS-2 mRNA expression was $0.48 \pm 0.03$ arbitrary units (a.u.) in the control group and $0.41 \pm 0.03$ a.u. in the AN-215-treated group, the decrease of 14% being not significant. The levels of BRS-3 were $0.72 \pm 0.04$ a.u. in the control and $0.45 \pm 0.04$ a.u. in the AN-215-treated group corresponding to a 31% reduction ($P < 0.05$).

### DISCUSSION

Although some progress has been made in the treatment of SCLC, the overall therapeutic results are not satisfactory and the development of novel treatment modalities is required. In the present study we evaluated the antitumor effects of targeted cytotoxic bombesin analogue AN-215, in nude mice bearing xenografts of the human SCLC cell line H-69. A single i.v. injection of 200 nmol kg$^{-1}$ AN-215 inhibited significantly the growth of NCI-H-69 tumours for at least 28 days and caused the death of only one of six animals. In contrast, treatment with AN-201 or a mixture of AN-201 and RC-3094 was ineffective and induced 71–83% mortality among experimental animals within 4 weeks after the injection. The group treated with the carrier peptide showed no inhibition of the tumour growth.

Histological evaluation revealed that tumours from mice treated with AN-215 showed increased apoptotic index and more extensive necrosis as compared to the control group.

High binding affinity of the receptor ligand is required for efficient targeting of the tumour. The cytotoxic bombesin analogue AN-215 has been shown to displace binding of [$^{125}$I-Tyr$^4$] bombesin to receptors on Swiss 3T3 cells at nanomolar concentrations with a $K_i$ value of 1.6 nM (Nagy et al, 1997). Previously we demonstrated that isolated membrane fractions of H-69 tumour cells growing in nude mice display high-affinity binding for [$^{125}$I-Tyr$^4$]bombesin (Pinski et al, 1994; Koppán et al, 1998). In this study, molecular biology analysis at the mRNA level showed that H-69 tumours express mRNA for BRS-2 and BRS-3 but not for BRS-1. It has been suggested that the pharmacological properties of BRS-3 are distinct from those of BRS-1 and BRS-2 (Mantey et al, 1997). Interestingly, the tumours from the AN-215-treated group expressed lower levels of BRS-3 as compared to the controls. This could be the consequence of tumour inhibition or a direct result of the interaction between the cytotoxic bombesin analogue and the BRS-3 receptor. Considering that the treatment consisted of a single i.v. injection of the cytotoxic analogue 28 days before the mRNA analysis, the down-regulation of the BRS-3 may be interpreted as a consequence of the tumour inhibition rather than a direct result of the interaction between the cytotoxic bombesin analogue and the BRS-3 receptor. A down-regulation of BRS-3 due to endocrine effects is unlikely because a low dose of the bombesin analogue AN-215 was used in a magnitude of nanomoles. Thus, it is more likely that the down-regulation of BRS-3 is a result of the suppression of the tumour growth, implicating BRS-3 as well as its unknown natural ligand in the autocrine stimulation of the H-69 SCLC. However, these findings regarding the role of BRS-3 in the growth H-69 SCLC should be confirmed by more detailed studies. Furthermore, an interaction of the cytotoxic bombesin analogue AN-215 with receptors other than the three known subtypes of bombesin receptors should be also considered as a possibility (Nagalla et al, 1995; Orosz et al, 1995).

In receptor-targeted chemotherapy, the cytotoxic moiety must retain the anti-tumour activity after conjugation to the carrier peptide. We have previously demonstrated (Nagy et al, 1996) that AN-215 preserves the cytotoxic activity of AN-201 in vitro. 2-Pyrrolino-DOX (AN-201) is a daunosamine-modified derivative of DOX which is 500–1000 times more potent than DOX in vitro. In vitro, AN-201 inhibited growth of cultured NCI-H-69 cells faster than AN-215 (Figure 3). This is probably due to differences in the mechanism of action of these cytotoxic agents. AN-201 could enter the cells by passive diffusion, as described for DOX, while AN-215 may require an interaction with the bombesin receptor in order to exert its cytotoxicity. In vitro, a difference in the cytotoxicity between AN-201 and AN-215 could be demonstrated at $10^{-8}$ M. Addition of $10^{-9}$ M AN-201 had no effect, and $10^{-7}$ M AN-201 caused the death of approximately 60% of cells within 1 min of treatment. If the delay in the effect of AN-215 as compared to AN-201 at a concentration of $10^{-8}$ M was due to the

 © 1999 Cancer Research Campaign deconjugation of the cytotoxic radical AN-201, we should have also observed a retardation in the action of AN-215 at a dose of $10^{-7}$ M. In addition, in co-cultured H-69 SCLC and H-157 non-SCLC cells that do not express bombesin receptors, AN-215 was targeted selectively to the bombesin receptors-positive H-69 SCLC cell line (Kiaris and Schally, in preparation).

Dose-limiting toxicity for AN-201 is essentially based on the haematopoietic toxicity, and the maximum tolerated dose in male nude mice is between 175 and 200 nmol $kg^{-1}$ (Plonowski, et al, 1999). In this study AN-215 was less toxic than AN-201, but its maximum tolerated dose was similar to that of AN-201, about 200 nmol $kg^{-1}$. In contrast, 300 nmol $kg^{-1}$ of AN-215 was well-tolerated by Copenhagen rats in a most recent study (A Plonowski et al, unpublished data), while 115–125 nmol $kg^{-1}$ of AN-201 was lethal (Schally and Nagy, 1999). A very similar toxicity pattern was found in the case of our cytotoxic somatostatin analogue AN-238 containing AN-201. Because AN-201 is linked to the peptide carriers in these analogues through an ester bond, hydrolytic cleavage of these conjugates by carboxylate esterase enzymes (EC 3.1.1.1) can occur in blood and in target tissues. These enzymes are ubiquitous and show low substrate specificity. The half-lives of cytotoxic peptide conjugates containing DOX-14-*O*-hemiglutarate or 2-pyrrolino-DOX-14-*O*-hemiglutarate such as AN-215 in serum of mice, rats and humans were found to be about 10, 30 and 120 min respectively (Plonowski et al, 1999). A better tolerance of AN-215 by rats than by mice as compared to AN-201 may be due to the lower esterase activity in rats than in mice. A less-pronounced esterase activity in blood may allow a more effective targeting of the cytotoxic conjugate to receptor-positive tissue. Besides the tumour cells, various normal, non-cancerous cells in the central nervous system, lung, or the gastrointestinal tract express high levels of receptors for bombesin. As our previous studies on the toxicity and efficacy of cytotoxic somatostatin analogue AN-238 indicate, these cells may not be irreversibly damaged by the DNA-intercalating antiproliferative agent, 2-pyrrolino-DOX (AN-201), or the resting cells may be able to reproduce the damaged cells (Plonowski et al, 1999). Because the esterase enzyme activity is lower in human beings than in rodents, an even better targeting of the cytotoxic agent to receptor-positive tissue can be expected in humans.

This is the first report on the anti-tumour effects of targeted cytotoxic analogue of bombesin AN-215 in human SCLC-derived cell line. Our results demonstrate that in nude mice bearing xenografts of H-69 SCLC, cytotoxic bombesin analogue AN-215 shows higher tumour inhibitory activity and lower toxicity than its cytotoxic moiety AN-201. Our results suggest that tumours expressing bombesin receptors, such as SCLC, could be treated using targeted cytotoxic bombesin analogues.

### ACKNOWLEDGEMENTS

We thank Harold Valerio and Veronica Lea for technical assistance. The work described in this paper was supported by the Medical Research Service of the Veterans Affairs Department and a grant from ASTA Medica (Frankfurt am Main, Germany) to Tulane University (all to AVS).

### REFERENCES

Cuttitta F, Carney DN, Mulshine J, Moody TW, Fedorko J, Fischler A and Minna JD (1985) Bombesin-like peptides can function as autocrine growth factors in human small-cell lung cancer. *Nature (London)* **316**: 823–826

Draoui M, Chung P, Park M, Birrer M, Jakowlew S and Moody TW (1995) Bombesin stimulates c-*fos* and c-*jun* mRNAs in small cell lung cancer cells. *Peptides* **16**: 289–292

Fathi Z, Corjay MH, Shapira J, Wada E, Benya R, Jenesen R, Viallet J, Sausville EA and Battey JF (1993) BRS-3: a novel bombesin receptor subtype selectively expressed in testis and lung carcinoma cells. *J Biol Chem* **268**: 5979–5984

Ihde DC (1995) Small cell lung cancer. *Chest* **107**: 243–248

Koppán M, Halmos G, Arencibia JM, Lamharzi N and Schally AV (1998) Bombesin/gastrin-releasing peptide antagonists RC-3095 and RC-3940-II inhibit tumor growth and decrease the levels and mRNA expression of epidermal growth factor receptors in H-69 small cell lung cancer. *Cancer* **83**: 1335–1343

Mantey SA, Weber HC, Sainz E, Akeson M, Ryan RR, Prahdan TK, Searles RP, Spindel ER, Battey JF, Coy DH and Jensen RT (1997) Discovery of a high affinity radioligand for the human orphan receptor, bombesin receptor subtype 3, which demonstrates that it has a unique pharmacology compared with other mammalian bombesin receptors. *J Biol Chem* **272**: 26062–26071

Moody TW and Cuttitta F (1993) Growth factor and peptide receptors in small cell lung cancer. *Life Sci* **52**: 1161–1173

Moody TW, Carney DN, Cuttitta F, Quattrocchi K and Minna JD (1985) High affinity receptors for bombesin/GRP-like peptides on human small cell lung cancer. *Life Sci* **37**: 105–113

Nagalla SR, Barry BJ, Creswick KC, Eden P, Taylor JT and Spindel E (1995) Cloning of a receptor for amphibian [$Phe^{13}$]bombesin distinct for the receptor for gastrin-releasing peptide: identification of a forth bombesin receptor subtype (BB4). *Proc Natl Acad Sci USA* **92**: 6205–6209

Nagy A, Armatis P and Schally AV (1996) High yield conversion of doxorubicin to 2-pyrrolinodoxorubicin, an analog 500–1000 times more potent: structure–activity relationship of daunosamine-modified derivatives of doxorubicin. *Proc Natl Acad Sci USA* **93**: 2464–2469

Nagy A, Armatis P, Cai R-Z, Szepeshazi K, Halmos G and Schally AV (1997) Design, synthesis, and in vitro evaluation of cytotoxic analogs of bombesin-like peptides containing doxorubicin or its intensely potent derivative, 2-pyrrolinodoxorubicin. *Proc Natl Acad Sci USA* **94**: 652–656

Orosz A, Schrett J, Nagy J, Bartha L, Schon I and Nyeki O (1995) New short-chain analogs of a substance-P antagonist inhibit proliferation of human small-cell lung-cancer cells in vitro and in vivo. *Int J Cancer* **60**: 82–877

Pastan I and FitzGerald D (1991) Recombinant toxins for cancer treatment. *Science* **254**: 1173–1177

Pinski J, Schally AV, Halmos G, Szepeshazi K, Groot K, O'Byrne K and Cai R-Z (1994) Effects of somatostatin analogue RC-160 and bombesin/gastrin releasing peptide antagonists on the growth of human small-cell and non-small-cell lung carcinomas in nude mice. *Br J Cancer* **70**: 886–892

Plonowski A, Schally AV, Nagy A, Sun B and Szepeshazi K (1999) Inhibition of PC-3 human androgen-independent prostate cancer and its metastases by cytotoxic somatostatin analog AN-238. *Cancer Res.* **59**: 1947–1953

Plumb JA, Milroy R and Kaye SB (1989) Effects of the pH dependence of 3-(4,5-Dimethylthiazol-2yl)-2, 5-diphenyltetrazolium bromide-formazan absorption on chemosensitivity determined by a novel tetrazolium-based assay. *Cancer Res* **49**: 4435–4440

Schally AV and Nagy A (1999) Chemotherapy targeted to hormone receptors on tumors. *Eur J Endocrinol* **141**: 1–14

Spindel ER, Giladi E, Segerson TP and Nagalla S (1993) Bombesin-like peptides: of ligands and receptors. *Recent Prog Horm Res* **48**: 365–391

Szepeshazi K, Milovanovic S, Lapis K, Groot K and Schally AV (1992) Growth inhibition of estrogen independent MXT mouse mammary carcinomas in mice treated with an agonist or antagonist of LHRH, an analog of somatostatin or a combination. *Breast Cancer Res Treat* **21**: 181–192

Toi-Scott M, Jones CL and Kane MA (1996) Clinical correlates of bombesin-like peptide receptor subtype expression in human lung cancer cells. *Lung Cancer* **15**: 341–354

UKCCCR (1998) United Kingdom Coordinating Committee on Cancer Research (UKCCCR) guidelines for the welfare of animals in experimental neoplasia, 2nd edn. *Br J Cancer* **77**: 1–10

Von Schrenck T, Heinz-Erian P, Moran T, Mantey SA, Gardner JD and Jenson RT (1989) Neuromedin B receptor in esophagus: evidence for subtypes of bombesin receptors. *Am J Physiol* **256**: 747–758

© 1999 Cancer Research Campaign 
